# Lexical repertoire of 24 and 30-month-old children speaking Brazilian portuguese: preliminary results

**DOI:** 10.1590/2317-1782/20242023268en

**Published:** 2024-05-20

**Authors:** Carolina Felix Providello, Ana Paola Nicolielo Carrilho, Vânia Peixoto, Maria de Fátima Serdoura Cardoso Maia, Simone Rocha de Vasconcellos Hage

**Affiliations:** 1 Departamento de Fonoaudiologia, Faculdade de Odontologia de Bauru – FOB, Universidade de São Paulo – USP - Bauru (SP), Brasil.; 2 Faculdade de Odontologia – FO, Universidade de São Paulo – USP - São Paulo (SP), Brasil.; 3 Escola Superior de Saúde Fernando Pessoa, Universidade Fernando Pessoa - Porto, Portugal.

**Keywords:** Child Development, Language Development, Child Language, Vocabulary, Verbal Learning

## Abstract

**Purpose:**

To check the lexical repertoire of Brazilian Portuguese-speaking children at 24 and 30 months of age and the association between the number of words spoken and the following variables: socioeconomic status, parents’ education, presence of siblings in the family, whether or not they attend school, and excessive use of tablets and cell phones.

**Methods:**

30 parents of children aged 24 months living in the state of São Paulo participated in the study. Using videoconferencing platforms, they underwent a speech-language pathology anamnesis, an interview with social services, and then they completed the “MacArthur Communicative Development Inventory - First Words and Gestures” as soon as their children were 24 and 30 months old. Quantitative and qualitative inferential inductive statistics were applied.

**Results:**

the median number of words produced was 283 at 24 months and 401 at 30 months, indicating an increase of around 118 words after six months. The child attending a school environment had a significant relationship with increased vocabulary.

**Conclusion:**

The study reinforces the fact that vocabulary grows with age and corroborates the fact that children aged 24 months already have a repertoire greater than 50 words. Those who attend school every day produce at least 70 more words than those who do not.

## INTRODUCTION

Before the pandemic period, the Centers for Disease Control and Prevention (CDC), the United States public health agency, reviewed several developmental milestones, including language. The revisions changed the clinical manual for parents and caregivers of young children, entitled “Learn the Signs. Act Early.” and were published by the Pediatrics journal^(1)^. One of the milestones that was changed was the number of words produced by the children. Initially, the manual indicated that 24-month-old children should produce around 50 words. With the review, the expectation of producing the same number of words was increased to 30 months. The change was based on standardized scales and experimental studies^(2-5)^.

One of the references mentioned to justify the change was the “Capute” scale^(2)^, a standardized assessment tool that identifies delays, deviations and discrepancies between areas of development. According to the scale, children are expected to have 100 words by the age of 24 months, without stating the exact expectation at 24. Another reference was the description of a prototype tool for evaluating child development indicators, which used data from more than 21,000 children assessed in 10 low/middle-income countries. For the age group between 24 and 30 months, the prototype describes that children use modifying terms (adjectives, pronouns, adverbs), but there is no specification regarding the number of words produced at these ages^(3)^. A longitudinal study that followed 40 mother-child dyads was also mentioned to support the change. Three language indices were examined (vocal imitations, first words produced spontaneously and receptive language) and evaluated as to how well they could predict three significant language milestones of the second year of life: production of 50 words, combination of words, and use of language to express a memory. The findings contributed to generating and testing models to explain the variation in child language development in the second year of life, but there is no description of the number of words produced at 24 months^(4)^. The “Ages & Stages Questionnaires - ASQ–3” screening test^(5)^ states that children should speak three or more words between nine and ten months, four at 14 months, eight at 16 months and 15 or more at 22 months, as well as “daddy” and “mommy”. At 24 and 30 months, items are presented for verification, namely, verbal comprehension, imitation, spontaneous production of sentences of two or more words, production of possessive pronouns, naming body parts and ability to describe pictures or tell facts. The items make up the topic of “communication” and there is no mention of the number of words spoken in either of these two age groups.

In addition to the scale, screening tool and two experimental articles, the support for change was also based on data presented in materials designed for caregivers of young children, without specific milestones on the number of words a child should speak at 24 months.

The lack of specific information on the number of words in the texts used by the CDC to support the change, the note issued by ASHA (American Speech-Language-Hearing Association)^(6)^ in 2022 expressing concern about the postponement of some language milestones, and the scarcity of studies on the topic with Portuguese-speaking babies drove us to this study.

In this context, the objective is to check the lexical repertoire of Brazilian Portuguese-speaking children at 24 and 30 months, and the association between the number of words spoken and the following variables: socioeconomic status, parents’ education, presence of siblings in the family, whether or not they attend school, and excessive use of tablets and cell phones. Our hypothesis is that the 50-word milestone at 30 months is late and may lead families to delay the referral of children at risk of language development disorders.

## METHOD

This study was approved by the Human Research Ethics Committee of the Bauru School of Dentistry of the University of São Paulo (CAAE: 59714422.0.0000.5417) and opinion number 6.646.588. Information on the procedures to be carried out was provided by means of the Free and Informed Consent Form (FICF), and acceptance was given by signing the form remotely, in accordance with resolution no. 466 of 12/12/2012.

### Participants

Parents of children aged 24 months (which could vary between 22 and 26 months) living in the state of São Paulo were invited to take part in the study via social media. Those interested filled in the Google Forms containing the ICF and a field to fill in the child’s date of birth and state of origin. Those who signed the ICF and were of the required age and state to participate in the study advanced to the other stages of the study. At the end of all stages, 30 parents met the four inclusion criteria:

1. Living in the state of São Paulo with a child between 22 and 26 months, with a lexical repertoire of at least three words, in addition to “daddy” and “mommy”;^(1)^
2. Children without signs or multidisciplinary diagnosis of development disorder, including language, such as genetic or neurological syndromes, hearing impairment, autism spectrum disorder or degenerative diseases;
3. Children who did not undergo any intervention process to stimulate development, such as speech therapy, psychological or occupational therapy;
4. Complete all stages of the study.

### Procedures

Step 1 - Using video conferencing platforms (Google Meet or Zoom) or video call via WhatsApp, parents underwent a speech therapy anamnesis used at the school clinic to treat children with communication, speech and language complaints. The data obtained included parents’ education, family’s social and recreational activity, presence of siblings in the family, child's school attendance, and biological risk factors for child development. In order to meet the inclusion criteria, it was also investigated whether the child was or had been undergoing any kind of intervention, whether they had sensory deficits or any neurodevelopmental disorders that had already been identified by a health professional or were under investigation. Home videos of the children were requested to complement the interview. If any of these conditions were confirmed, the child was excluded from the study and the family was advised on stimulation strategies or to seek multi-professional assessment/intervention.

Step 2 - Those who met the inclusion criteria from one to three were subjected to a socioeconomic classification interview, using an instrument that covers five indicators (economic situation, number of family members, education of family members, housing and occupation / work of family members) that converge into a score and social stratification: Lower Low (LL), Upper Low (UL), Lower Middle (L), Middle (M), Upper Middle (UM) and High (H)^(7)^. The instrument was scored and analyzed by a social worker from the Higher Education Institution.

Step 3 - Once the previous steps were completed, the parents received the vocabulary list of the systematized protocol for communicative development “MacArthur Inventory of Communicative Development First Words and Gestures”^(8)^, which contains 421 words distributed in 22 semantic categories. The list was made available on a Google Forms form after explanations were given on how to complete it via videoconference.

Step 4 - The inventory was reapplied six months after the first application when the children were 30 months old, which could vary between 28 and 32 months, depending on the child’s age at the first application. It is important to highlight that there is no impediment to the protocol being applied at an older age when the purpose is to compare age groups.

### Statistical analysis

Quantitative and qualitative inferential inductive statistics were used. The Shapiro-Wilk test was used to compare independent samples in order to verify the type of distribution (normal or non-normal), and the Wilcoxon Signed Rank test was used to calculate the sample using the median, 1st and 3rd quartile (non-normal distribution). The minimum and maximum values describe the lowest and highest values of the data found. For the qualitative analysis, classification by percentage was carried out to characterize the data obtained in the anamnesis, and bivariate linear regression analysis was carried out to check the association between the outcome (number of words) and the predictors (socioeconomic status, parents’ education, etc.).

## RESULTS

A total of 30 parents met all the inclusion criteria out of the 60 who showed interest in participating in the study, and 66.7% of the children whose parents responded to the inventory were female.

**Figure 1:**
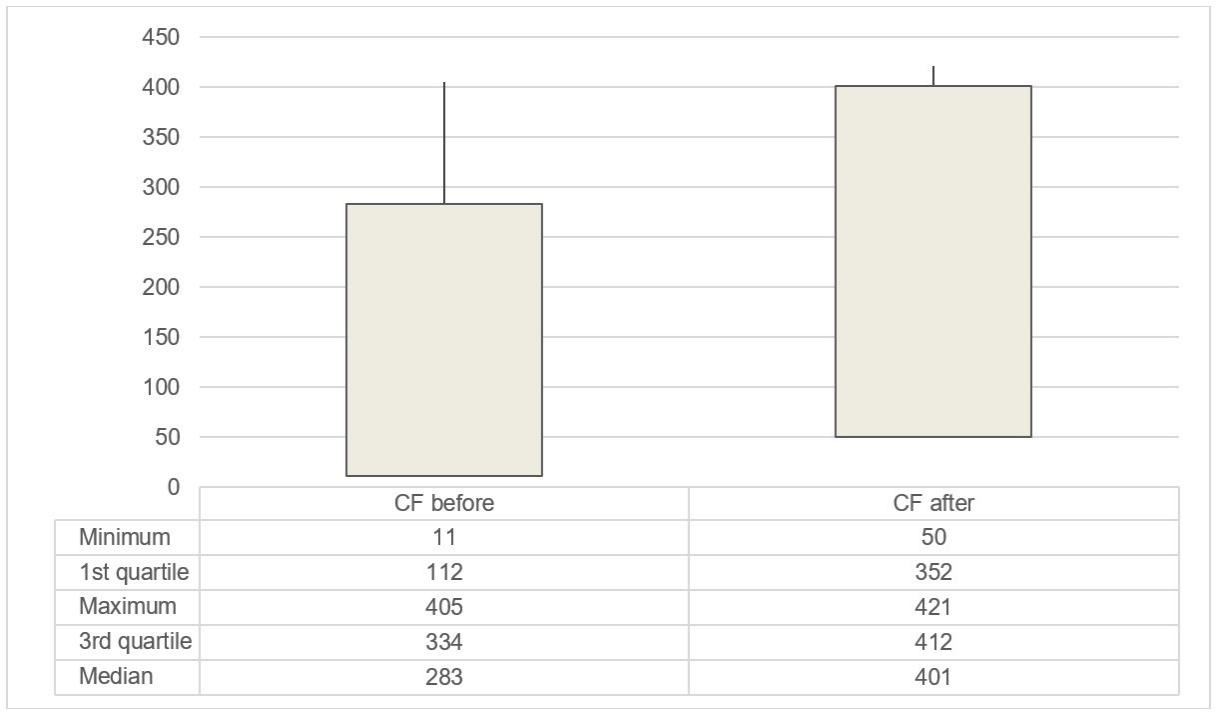
Median, 1st and 3rd quartile of the number of words children speak (expressive vocabulary) at 24 months and 30 months

Figure 1 shows the median, 1st and 3rd quartile of the number of words the children speak (expressive vocabulary) at 24 months and 30 months.

Statistical test: Jamovi (Version 2.3) – Wilcoxon. Caption: CF = understands and speaks; CF before = number of words produced at 24 months; CF after = number of words produced at 30 months

The average number of words spoken by children aged 24 and 30 months was calculated using the median, which shows a central value based on a non-normal distribution, avoiding conceptual statistical error.

The speech-therapy interview and the social service interview provided data on environmental variables. Regarding socioeconomic status, 73.3% of the sample had a lower middle socioeconomic status and 26.7% had an upper low socioeconomic status. As for education, 80% of mothers and 66.7% of fathers had higher education and 100% of the sample finished high school. Most of the children attend school (73.3%), 60.0% have siblings and 11.1% use tablets/mobile phones for more than two hours a day.

Associations were made between the lexical repertoire (number of words spoken) and the following variables: family’s socioeconomic status, parents’ education, siblings in the family and school attendance. Statistical analysis was carried out using bivariate linear regression and “attend school” showed a significant relationship among the four variables, as shown in Table 1.

**Table 1:** Values of p from simple or bivariate linear regression of proposed associations

**Associations**	**p**	**Interpretation**
1. Family’s socioeconomic status	0.680	p>0.2 –NR
2. Parents’ education	0.814	p>0.2 –NR
3. Siblings in the family	0.773	p>0.2 –NR
**4. School attendance**	**0.044**	**p<0.2 – R**
5. Screen time > 2 hours	0.241	p>0.2 –NR

Statistical test: Jamovi (Version 2.3) - Simple or bivariate linear regression analysis

Caption: p = value of relationship between variables; p>0.2 – NR = independent variable not directly related to dependent variable; p<0.2 – R = independent variable directly related to dependent variable

## DISCUSSION

This study investigated the lexical repertoire of Brazilian Portuguese-speaking children at 24 and 30 months and examined the existence of an association between the number of words produced by the children and certain predictors.

The median number of words spoken was 283 at 24 months and 401 at 30 months, indicating an increase of around 118 words after 6 months (Figure 1). Data from different languages show that the 50-word milestone can be reached even before 24 months. The vocabulary of 51 Slovenian-speaking children was monitored from 16 to 31 months at six different times using a vocabulary checklist containing 680 words. The results indicated that children produce an average of 296 words at 25 months, with a vocabulary explosion occurring between 16 and 22 months^(9)^. A study carried out with 504 Catalan-speaking children between 10 and 18 months investigated which factors best explained the acquisition of initial expressive vocabulary. The data were obtained using the MacArthur-Bates Communicative Development Inventory^(8)^. It was noted that the process of vocabulary acquisition began slowly, gradually increasing until 16 months, the age at which the lexical explosion was observed. From this age, in two months, the vocabulary increased from 20 to more than 60 words at 18 months^(10)^.

A study followed 329 children in the US metropolitan area between 2 and 36 months and 9 children between 38 and 47 months, using software associated with the McArthur Inventory. It found that they produced 25 words between 12 and 17 months, 177 words between 18 and 24 months, and 455 words above 25 months^(11)^. Another study followed 2,084 European Portuguese-speaking children who were assessed longitudinally at 16, 21, 25 and 30 months. The size of the lexicon was 28 words at 16 months and reached 415 words at 30 months^(12)^. A longitudinal follow-up carried out with 92 Kenyan children between eight and 24 months, using a vocabulary protocol adapted to the region, language, social conditions in the area and parents’ education, showed that the average number of words produced was 19 by 15 months and 23 by 30 months, indicating a delay in word production^(13)^. It is worth noting that the context of social vulnerability may have influenced the results.

Thus, investigations with children of different languages reinforce the fact that vocabulary grows with age and corroborates the fact that children at 24 months have a repertoire of more than 50 words. The postponement of the 50-word milestone to 30 months proposed by the CDC could lead families to delay the referral of children at risk of language development disorders.

Regarding the verification of predictors relating to socioeconomic status, the preliminary results showed that 73.3% of the 30 parents interviewed had a lower middle socioeconomic status and 26.7% had an upper low socioeconomic status, indicating a socio-family reality equivalent to classes B and C, similar to that of the majority of the population living in the South and Southeast regions of Brazil, according to the Brazil criterion. The mothers’ education was higher than the fathers’. A study carried out in 2018 analyzing data from 2084 European Portuguese-speaking children between 16 and 30 months indicated that the number of words produced by children over 24 months was significantly related to maternal education, as children of more educated mothers produced more words in general, as well as more social terms, common nouns, predicates and function words^(12)^. The higher the parents’ level of education and information, the greater the verbal interaction with their children, allowing for greater language development. Considering the sample studied, 73.3% of the children attended school and this variable showed a statistically significant relationship (Table 1). Those who attend school daily produce at least 70 more words than those who don't. Being inserted in a school environment can reverse the impact of socioeconomic status on the development of the semantic aspect, as the range of vocabulary is related to the number of lexical entries that a child has through incidental learning during repeated exposure^(9)^. In a study that investigated the extent to which pre-school language environments are associated with children’s vocabulary skills, the findings highlighted close associations between the conversational interactions children have with teachers^(14)^. Although this was a study with older children (four years old), it reinforces that certain classroom activities can better promote conversational interactions between children and teachers. It is critical that school contexts are considered in the debate on word acquisition, as there is a lack of studies that relate child development to school environment^(15)^.

## CONCLUSION

The data obtained indicate that children at 24 months have well over 50 words, around 283 lexical items and around 401 at 30 months, similar to the data obtained in studies of other languages. There is an association between the lexical repertoire and the fact that the child attends school, with an increase of around 70 words for children who are within the school environment. Other relationships such as socioeconomic status, parents’ education, presence of siblings and the use of screens may be important predictors, but at this point in the study, there was no association. The study is being replicated with European Portuguese-speaking children.
