# Supplementary material for: Lexical repertoire of 24 and 30-month-old children speaking Brazilian portuguese: preliminary results
Source: Codas. 2024 May 20;36(4):e20230268. doi: 10.1590/2317-1782/20242023268en (PMC11129851; doi:10.1590/2317-1782/20242023268en)
Supplement: Translation

# Translation: Repertório lexical de crianças de 24 e 30 meses falantes do português brasileiro: resultados preliminares

**DOI:** 10.1590/2317-1782/20242023268pt

**Authors:** Carolina Felix Providello, Ana Paola Nicolielo Carrilho, Vânia Peixoto, Maria de Fátima Serdoura Cardoso Maia, Simone Rocha de Vasconcellos Hage

**Affiliations:** 1 Departamento de Fonoaudiologia, Faculdade de Odontologia de Bauru – FOB, Universidade de São Paulo – USP - Bauru (SP), Brasil.; 2 Faculdade de Odontologia – FO, Universidade de São Paulo – USP - São Paulo (SP), Brasil.; 3 Escola Superior de Saúde Fernando Pessoa, Universidade Fernando Pessoa - Porto, Portugal.

**Keywords:** Desenvolvimento Infantil, Desenvolvimento da Linguagem, Linguagem Infantil, Vocabulário, Aprendizado Verbal

## Abstract

**Objetivo:**

Verificar o repertório lexical de crianças falantes do português brasileiro aos 24 e 30 meses e a associação entre a quantidade de palavras faladas e as variáveis: nível socioeconômico, escolaridade dos pais, presença de irmãos no convívio familiar, frequentar ou não escola e uso exacerbado de *tablets* e celulares pelas crianças.

**Método:**

30 pais de crianças com 24 meses, residentes no estado de São Paulo participaram do estudo. Por meio de plataformas de videoconferência eles foram submetidos à anamnese fonoaudiológica, entrevista com o serviço social e preencheram o “Inventário MacArthur de Desenvolvimento Comunicativo - Primeiras Palavras e Gestos”, quando seus filhos tinham 24 e 30 meses. Foi aplicada estatística indutiva inferencial, quantitativa e qualitativa.

**Resultados:**

A mediana das palavras emitidas foi de 283 aos 24 meses e 401 aos 30 meses, indicando aumento em torno de 118 palavras após seis meses. A criança estar frequentando ambiente escolar apresentou relação significativa com o aumento do vocabulário.

**Conclusão:**

O estudo reforça o crescimento do vocabulário conforme o avanço da idade e corrobora o fato de as crianças com 24 meses já possuírem um repertório maior que 50 palavras. Aqueles que frequentam escola diariamente produzem pelo menos 70 palavras a mais dos que não frequentam.

## INTRODUÇÃO

Antes do período pandêmico, o Centro de Desenvolvimento para o Controle de Doenças (CDC), órgão de vigilância em saúde nos Estados Unidos, revisou vários marcos do desenvolvimento, incluindo os de linguagem. As revisões alteraram o manual clínico para pais e cuidadores de crianças pequenas, intitulado “*Learn the Signs. Act Early.” (*“Aprenda com os sinais. Aja cedo”) e foram divulgadas pela revista Pediatrics^(1)^. Um dos marcos alterados foi quanto ao número de palavras produzidas pelas crianças. Inicialmente o manual apontava que crianças aos 24 meses deveriam produzir cerca de 50 palavras. Com a revisão, aumentou-se para 30 meses a expectativa de produção deste mesmo número de palavras. A mudança teve amparo em escalas padronizadas e estudos experimentais^(2-5)^.

Uma das referências citadas para justificar a mudança foi a escala *“Capute”* ^(2)^, ferramenta de avaliação normatizada que identifica atraso, desvio e discrepância entre áreas do desenvolvimento. Segundo a escala, é esperado que as crianças tenham 100 palavras acima dos 24 meses, sem afirmar exatamente a expectativa aos 24. Outra referência foi a descrição de protótipo de ferramenta para avaliação de indicadores de desenvolvimento infantil em que se utilizou dados de mais de 21.000 crianças avaliadas em 10 países de baixa/média renda. Para a faixa etária entre 24 e 30 meses, o protótipo descreve que as crianças fazem uso de termos modificadores (adjetivos, pronomes, advérbios), mas não há especificação da quantidade de vocábulos produzidos nessas idades^(3)^. Estudo longitudinal que acompanhou 40 díades mãe-criança também foi citado para apoiar a mudança. Três índices de linguagem foram examinados (imitações vocais, primeiras palavras produzidas espontaneamente e linguagem receptiva) e o quanto esses índices poderiam prever três marcos significativos de linguagem do segundo ano de vida: produção de 50 palavras, combinação de palavras e uso da linguagem para expressar uma memória. Os achados contribuíram para gerar e testar modelos para explicar a variação do desenvolvimento da linguagem infantil no segundo ano de vida, mas não há descrição do número de palavras produzidas aos 24 meses^(4)^. No teste de triagem “*Ages & Stages Questionnaires - ASQ–3*”^(5)^ é referido que as crianças devem falar três ou mais palavras entre nove e dez meses, quatro aos 14 meses, oito aos 16 e 15 ou mais aos 22 meses, além de “papai” e “mamãe”. Aos 24 e aos 30 meses são apresentados itens para verificação, a saber, compreensão verbal, imitação, produção espontânea de sentenças de duas ou mais palavras, produção de pronomes possessivos, nomeação de partes do corpo e habilidade em descrever figuras ou contar fatos. Os itens compõem o tópico “comunicação” e não há menção sobre o número de palavras ditas em nenhuma dessas duas faixas etárias.

Além da escala, da ferramenta triagem e dois artigos experimentais, a sustentação da mudança também se apoiou em dados apresentados por meio de materiais elaborados para cuidadores de crianças pequenas, sem marcos específicos sobre o número de palavras que uma criança deva falar aos 24 meses.

A falta de informações específicas quanto ao número de palavras nos textos utilizados pelo CDC para apoiar a mudança, a nota emitida pela ASHA (*American Speech-Language-Hearing Association*)^(6)^ em 2022 demonstrando preocupação com a postergação de alguns marcos de linguagem, e a escassez de estudos sobre o tema em bebês falantes em língua portuguesa nos direcionou para a pesquisa em tela.

Neste contexto, o objetivo é verificar o repertório lexical de crianças falantes do português brasileiro aos 24 e 30 meses, e ainda, analisar a existência de associação entre a quantidade de palavras faladas e determinadas variáveis: nível socioeconômico, escolaridade dos pais, presença de irmãos no convívio familiar, frequentar ou não escola e uso exacerbado de *tablets* e celulares pelas crianças. Nossa hipótese é a de que o marco de 50 palavras aos 30 meses é tardio e pode levar as famílias a atrasarem o encaminhamento de crianças com risco para alterações no desenvolvimento da linguagem.

## MÉTODO

Este estudo foi aprovado pelo Comitê de Ética em Pesquisa em Seres Humanos da Faculdade de Odontologia de Bauru da Universidade de São Paulo, CAAE: 59714422.0.0000.5417 e número de parecer 6.646.588. Foram fornecidas informações sobre os procedimentos a serem realizados por meio do Termo de Consentimento Livre e Esclarecido (TCLE), e o aceite se deu por meio da assinatura do termo de forma remota, conforme a resolução n^o.^ 466, de 12/12/2012.

### Participantes

Foram convidados a participar do estudo, por meio das mídias sociais, pais de crianças com 24 meses (que puderam variar entre 22 e 26 meses), residentes no estado de São Paulo. Os interessados preencheram formulário do “*Google Forms*” contendo o TCLE e campo para preencher a data de nascimento da criança e o estado de origem. Aqueles que assinaram o TCLE, e tinham a idade e pertenciam ao estado requisito para participar do estudo avançaram para outras etapas da pesquisa. Ao final de todas as etapas, 30 pais atenderam aos quatro critérios de inclusão:

1. Morar no estado de São Paulo com filho (a) entre 22 e 26 meses, com repertório lexical de no mínimo três palavras, além de “papai” e “mamãe”;^(1)^
2. Crianças sem sinais ou diagnóstico multidisciplinar de alteração no desenvolvimento, incluindo o de linguagem, como síndromes genéticas, neurológicas, deficiência auditiva, transtorno do espectro autista ou doenças degenerativas;
3. Crianças que não foram submetidas a qualquer processo de intervenção para estimulação do desenvolvimento, a saber, fonoaudiológico, psicológico ou de terapia ocupacional;
4. Concluir todas as etapas do estudo.

### Procedimentos

Etapa 1 - Por meio de plataformas de videoconferência (*Google Meet* ou *Zoom*) ou chamada de vídeo por *WhatsApp*, os pais foram submetidos à anamnese fonoaudiológica utilizada em clínica-escola no atendimento de crianças com queixa de comunicação, fala e linguagem. Os dados obtidos foram: escolaridade dos pais, atividade social e lúdica da família, existência de irmãos no convívio familiar, frequência da criança em escola, fatores de risco biológico para o desenvolvimento infantil. Para atender os critérios de inclusão, também foi averiguado se a criança estava ou esteve em algum tipo de intervenção, se tem déficit sensorial ou alguma condição no rol dos transtornos do neurodesenvolvimento já constatada por profissional da saúde ou sob suspeita. Foram solicitados vídeos caseiros das crianças como complementação da entrevista. Caso alguma destas condições tenha sido confirmada, a criança foi suprimida do estudo e a família orientada quanto a estratégias de estimulação ou a buscar avaliação/intervenção multiprofissional.

Etapa 2 - Aqueles que atenderam aos critérios de inclusão de um a três, foram submetidos à entrevista de classificação socioeconômica, por meio de instrumento que abrange cinco indicadores (situação econômica, número de membros, escolaridade dos membros da família, habitação e ocupação / trabalho dos integrantes) que convergem numa pontuação e estratificação social: Baixa Inferior (BI), Baixa Superior (BS), Média Inferior (MI), Média (M), Média Superior (MS) e Alta (A)^(7)^. A pontuação e análise do instrumento foi realizada por assistente social da Instituição de Ensino Superior.

Etapa 3 - Preenchida as etapas anteriores, foi entregue aos pais para preenchimento, a lista de vocabulário do protocolo sistematizado do desenvolvimento comunicativo “Inventário *MacArthur* de Desenvolvimento Comunicativo Primeiras Palavras e Gestos^” (8)^, que contém 421 palavras distribuídas em 22 categorias semânticas. A lista foi disponibilizada em formulário do *“Google Forms”* após explicações fornecidas sobre seu preenchimento por videoconferência.

Etapa 4 – O inventário foi reaplicado seis meses após a primeira aplicação quando as crianças tinham 30 meses, podendo variar entre 28 e 32 meses, dependendo da idade da criança na primeira aplicação. Importante destacar que não há impedimento do protocolo ser aplicado em idade superior ao previsto quando se tem como objetivo a comparação entre faixas etárias.

### Análise estatística

Utilizou-se estatística indutiva inferencial, quantitativa e qualitativa. Foi aplicado Teste de *Shapiro-Wilk* para comparar as amostragens independentes no intuito de verificar o tipo de distribuição (normal ou não normal), e o Teste de *Wilcoxon Signed Rank,* para o cálculo da amostra pela mediana, 1º e 3º quartil (distribuição não normal). Os valores de mínimo e máximo descrevem os valores menores e maiores dos dados encontrados. Para a análise qualitativa foi feita classificação por percentual para caracterização dos dados obtidos na anamnese e análise de regressão linear bivariada para verificar a associação entre o desfecho (número de palavras) e os preditores (nível socioeconômico, escolaridade dos pais etc.).

## RESULTADOS

30 pais atenderam a todos os critérios de inclusão dentre os 60 que demonstraram interesse em participar do estudo. Eram do sexo feminino 66,7% das crianças cujos pais responderam ao inventário.

A Figura 1 exibe a mediana, 1º e 3º quartil da quantidade de palavras que as crianças falam (vocabulário expressivo) aos 24 meses e aos 30 meses.

**Figura 1:** Mediana, 1º e 3º quartil da quantidade de palavras que as crianças falam (vocabulário expressivo) aos 24 meses e aos 30 meses

Teste estatístico: *Jamovi* (Versão 2.3) – Wilcoxon. Legenda: CF = compreende e fala; CF antes = número de palavras produzidas aos 24 meses; CF depois = número de palavras produzidas aos 30 meses

A média de palavras faladas por crianças com 24 e 30 meses foi calculada pela mediana que demonstra um valor central baseado em distribuição não normal, evitando erro estatístico conceitual.

A entrevista fonoaudiológica e a do serviço social forneceram dados sobre variáveis do ambiente. Referente ao nível socioeconômico, 73,3% da amostra tem nível socioeconômico médio inferior e 26,7% baixo superior. Quanto à escolaridade, 80% das mães e 66,7% dos pais têm nível superior e 100% da amostra completou o ensino médio. A maioria das crianças frequenta escola (73,3%), 60,0% têm irmãos e 11,1% das crianças fazem uso de *tablets*/celulares por mais de duas horas por dia.

Foram feitas associações entre o repertório lexical (número de palavras emitidas) e as variáveis: nível socioeconômico da família, escolaridade dos pais, existência de irmãos no convívio familiar e estar frequentando ambiente escolar. A análise estatística ocorreu por regressão linear bivariada sendo que das quatro variáveis, “frequentar escola” apresentou relação significativa, como indica a Tabela 1.

**Tabela 1:** Valores de p da regressão linear simples ou bivariada das associações propostas

**Associações**	p	Interpretação
1. NSE dos pais	0,680	p>0,2 –NR
2. Escolaridade dos pais	0,814	p>0,2 –NR
3. Possuir irmãos	0,773	p>0,2 –NR
**4. Frequentar escola**	**0,044**	**p<0,2 – R**
5. Uso de telas por + de 2 horas	0,241	p>0,2 –NR

Teste estatístico: *Jamovi* (Versão 2.3) – Análise de regressão linear simples ou bivariada

Legenda: NSE = nível socioeconômico; p = valor de relação entre as variáveis; p>0,2 – NR = variável independente não relacionada diretamente com a variável dependente; p<0,2 – R = variável independente relacionada diretamente com a variável dependente

## DISCUSSÃO

Este estudo verificou o repertório lexical de crianças falantes do português brasileiro aos 24 e aos 30 meses, e examinou a existência de associação entre a quantidade de palavras produzidas pelas crianças e determinados preditores.

A mediana das palavras emitidas foi de 283 aos 24 meses e 401 aos 30 meses, indicando aumento em torno de 118 palavras após 6 meses (Figura 1). Dados obtidos em diferentes línguas têm apontado que o marco de 50 palavras pode ser atingido até antes mesmo dos 24 meses. O vocabulário de 51 crianças de língua eslovena foi monitorado dos 16 aos 31 meses, em seis momentos diferentes por meio de lista de verificação de vocabulário contendo 680 palavras. Os resultados indicaram que crianças produzem em média 296 palavras com 25 meses, com explosão vocabular ocorrendo entre 16 e 22 meses^(9)^. Estudo feito com 504 crianças de língua catalã com idades entre 10 e 18 meses verificou quais fatores melhor explicavam a aquisição do vocabulário expressivo inicial. Os dados foram obtidos por meio do Inventário de Desenvolvimento Comunicativo *MacArthur-Bates* ^(8)^,. Observou-se que o processo de aquisição do vocabulário iniciou-se lentamente, aumentando gradativamente até os 16 meses, idade em que se observou a explosão lexical. A partir desta idade, em dois meses, o vocabulário aumentou de 20 para mais de 60 palavras aos 18 meses^(10)^.

Estudo acompanhou 329 crianças da região metropolitana norteamericana entre 2 e 36 meses e 9 entre 38 e 47 meses por meio de software associado ao Inventário *MacArthur*. Verificou-se que elas produziram 25 palavras dos 12 aos 17 meses, 177 palavras entre 18 e 24 meses e 455 acima dos 25 meses^(11)^. Outro experimento analisou 2.084 crianças falantes de português europeu avaliadas longitudinalmente aos 16, 21, 25 e 30 meses. O tamanho do léxico foi de 28 palavras aos 16 meses e chegou a 415 palavras aos 30^(12)^. Já um acompanhamento longitudinal realizado com 92 crianças quenianas de oito a 24 meses por meio de protocolo de vocabulário adaptado para a região, idioma, condições sociais do local e escolaridade dos pais respondentes, apontou que a média da produção de palavras foi 19 até 15 meses e de 23 aos 30 meses, indicando atraso^(13)^. Cabe ressaltar que o contexto de vulnerabilidade social pode ter influenciado os resultados.

Dessa forma, investigações com crianças de diferentes línguas reforçam o crescimento do vocabulário conforme o avanço da idade e corroboram o fato de as crianças com 24 meses possuírem um repertório maior que de 50 palavras. A postergação do marco de 50 palavras para 30 meses proposto pelo CDC pode levar as famílias a atrasarem o encaminhamento de crianças com risco para alterações no desenvolvimento da linguagem.

Em relação a verificação de preditores, referente ao nível socioeconômico, os resultados preliminares apontaram que 73,3% dos 30 pais entrevistados têm nível socioeconômico médio inferior e 26,7% baixo superior, indicando realidade sociofamiliar equivalente às classes B e C, semelhante ao da maioria da população residente nas regiões Sul e Sudeste do Brasil, conforme o critério Brasil. A escolaridade das mães se mostrou mais elevada que a dos pais. Pesquisa realizada em 2018 analisando dados de 2084 crianças falantes do português europeu com faixa etária de 16 a 30 meses indicou que o número de palavras produzidas pelas crianças acima de 24 meses, estava significativamente relacionado com a escolaridade materna, visto que filhos de mães mais escolarizadas produziram mais palavras em geral, bem como mais termos sociais, substantivos comuns, predicados e palavras funcionais^(12)^. Quanto maior o nível de escolaridade e informação dos pais maior a interação verbal com seus filhos permitindo maior desenvolvimento da linguagem. Da amostra estudada, 73,3% das crianças frequentam escola e esta variável apresentou relação estatística significativa (Tabela 1). Aqueles que frequentam escola diariamente produzem pelo menos 70 palavras a mais do que os que não frequentam. Estar inserido em ambiente escolar pode reverter o impacto da condição socioeconômica no desenvolvimento do aspecto semântico já que a amplitude de vocabulário está relacionada ao número de entradas lexicais que uma criança possui por meio da aprendizagem incidental, durante a exposição repetida^(9)^. Estudo em que se examinou até que ponto os ambientes linguísticos da pré-escola estão associados às habilidades de vocabulário das crianças, as descobertas sublinharam associações estreitas entre as interações conversacionais que as crianças têm com os professores^(14)^. Embora seja um estudo com crianças mais velhas (quatro anos), ele reforça que certas atividades em sala de aula podem promover melhor as interações conversacionais entre crianças e professores. É fundamental que contextos escolares sejam considerados no debate sobre aquisição de palavras, na medida que há carência de estudos que relacionem o desenvolvimento infantil com o ambiente escolar^(15)^.

## CONCLUSÃO

Os dados obtidos indicam que as crianças aos 24 meses apresentam número bem superior a 50 palavras, em torno de 283 itens lexicais e cerca de 401 aos 30 meses, semelhante aos dados obtidos em estudos de outras línguas. Evidencia-se associação entre o repertório lexical e o fato de a criança frequentar escola, observando-se um aumento em torno de 70 palavras para aquelas crianças que estão dentro do ambiente escolar. Outras relações quanto ao nível socioeconômico, escolaridade dos pais, possuir irmãos e uso de telas podem ser preditores importantes, mas neste momento da coleta, não demonstra associação. O estudo está sendo reproduzido com crianças falantes do português europeu.
